# Correction for Zhang et al., “MbovP0725, a secreted serine/threonine phosphatase, inhibits the host inflammatory response and affects metabolism in *Mycoplasma bovis*”

**DOI:** 10.1128/msystems.00570-24

**Published:** 2024-07-18

**Authors:** Hui Zhang, Yiqiu Zhang, Doukun Lu, Xi Chen, Yingyu Chen, Changmin Hu, Aizhen Guo

## AUTHOR CORRECTION

Volume 9, no. 4, e00891-23, http://doi.org/10.1128/msystems.00891-23. Page 21, Acknowledgments: “(#31661143015)” should read “(#32261143469).”

Page 21, Funding table, line 2: “31661143015” should read “32261143469.”

