# Supplementary material for: MbovP0725, a secreted serine/threonine phosphatase, inhibits the host inflammatory response and affects metabolism in Mycoplasma bovis
Source: mSystems. 2024 Mar 5;9(4):e00891-23. doi: 10.1128/msystems.00891-23 (PMC11019793; doi:10.1128/msystems.00891-23)
Supplement: Table S1 — Primers. [file msystems.00891-23-s0001.docx]

Table S1 Primers used for qRT-PCR and Q5 point mutation assay

| qRT-PCR | Primer Names | Forward sequences (5’-3’) | Reverse sequences (5’-3’) |
| --- | --- | --- | --- |
|  | IL-1β | TTCCATATTCCTCTTGGGGTAGA | AAATGAACCGAGAAGTGGTGTT |
|  | TNF-α | TCTTCTCAAGCCTCAAGTAACAAGC | CCATGAGGGCATTGGCATAC |
|  | IL-6 | CAGCAGGTCAGTGTTTGTGG | CTGGGTTCAATCAGGCGAT |
|  | β-actin | AGATCAAGATCATCGCGCCC | TAACGCAGCTAACAGTCCGC |
|  | Mbov_0002 | GGCCTTTGCAGCAAGTAACG | TGGTGCATCTGCTGGAAGTAA |
|  | Mbov_0062 | GACCCAGCACTTTTGCCTTG | CTTACCAGCCGGAGCAGAAA |
|  | Mbov_0131 | ATACTTTTCTGGCGCTGGCT | TTTTGGCTTTTTCTGCGCCA |
|  | Mbov_0155 | TGGCAAAATAAAAGGCGGAGC | CAACAGCAGAAAGCTTGCCA |
|  | Mbov_0273 | TGGTCAAATTACGCAGAAGCC | AGACCTGCAACAGCCATAGA |
|  | Mbov_0312 | ATGCTCTCCCTACTGGGCAT | TGCACCCATACCAACTGGAC |
|  | Mbov_0434 | AAAGGCGCAGCTTGTACAGT | TAGCCAACTGGCCCTTGAAC |
|  | Mbov_0482 | CGGTGTTGCAAATGTGCCTT | GTCATAACACCTTTGCCGCC |
|  | Mbov_0502 | ATTGCAGGTGCTGGTAGTGG | ATTTCAGCTGTGCCCTTGTT |
|  | Mbov_0565 | TGGCTGCCGCTGTACATTAT | AGCGCCATGTTCGCCTAATA |
|  | Mbov_0567 | TGATAGTGCTGTTGGTGGCT | CTGCACTAGGTTTTTGCACTGT |
|  | Mbov_0568 | CCACTGCACACTCCACCATT | GACTTAGGCCATGGTAGCCAA |
|  | Mbov_0668 | TGCAGGTGTAAGCGCTATGT | CACATTTGCATAAGCCGCCA |
|  | Mbov_0669 | TTGGTGGCGGAATGGCTTAT | AAACAGGCTCGACATCAGCA |
|  | Mbov_0769 | TGATGCCGGGATGCCATTAG | GCCCACTAAGTGCAAATGCT |
|  | Mbov_0774 | CCACAACCGACCCACAAAAG | AGCATCACGCATACTCCCTG |
|  | Mbov_0776 | GTCGGACACACTGGCAATCT | GCTTAGTGGCTGGACTGCTA |
|  | Mbov_0799 | TTGTGTTCAAGGGCGGTTTTC | TTCCATCGATGTCATCAGCGT |
|  | Mbov_0800 | ACAGTGGCTATAATGCAAAAGGC | TTCGCTATCTGTTTTCTTGCGT |
| Q5 point mutation | Mbov_0725-D15A | ATCTTCTTCGCACTGGACGGCAC | ATATTTAATGTCGTTCAGGTTG |
|  | Mbov_0725-T48A | GGTTCTGAATGCAGGCAGACCGT | ACCTGATGGGTCTTATTCAG |
|  | Mbov_0725-K210A | AGGTAGCGATGCAGGTACAGCAATTG | TTGGGCATCACATCCAGA |
|  | Mbov_0725-D236A | ATGTTTGGAGCAGCAGCAAATGATATTC | CAGGCTGTAATCGTAGATC |
